# MiR-200c Regulates Noxa Expression and Sensitivity to Proteasomal Inhibitors

**DOI:** 10.1371/journal.pone.0036490

**Published:** 2012-05-15

**Authors:** Mikael Lerner, Moritz Haneklaus, Masako Harada, Dan Grandér

**Affiliations:** Department of Oncology-Pathology, Cancer Center Karolinska (CCK), Karolinska Institutet, Stockholm, Sweden; The Chinese University of Hong Kong, Hong Kong

## Abstract

The pro-apoptotic p53 target Noxa is a BH3-only protein that antagonizes the function of selected anti-apoptotic Bcl-2 family members. While much is known regarding the transcriptional regulation of Noxa, its posttranscriptional regulation remains relatively unstudied. In this study, we therefore investigated whether Noxa is regulated by microRNAs. Using a screen combining luciferase reporters, bioinformatic target prediction analysis and microRNA expression profiling, we identified miR-200c as a negative regulator of Noxa expression. MiR-200c was shown to repress basal expression of Noxa, as well as Noxa expression induced by various stimuli, including proteasomal inhibition. Luciferase reporter experiments furthermore defined one miR-200c target site in the Noxa 3′UTR that is essential for this direct regulation. In spite of the miR-200c:Noxa interaction, miR-200c overexpression led to increased sensitivity to the clinically used proteasomal inhibitor bortezomib in several cell lines. This apparently contradictory finding was reconciled by the fact that in cells devoid of Noxa expression, miR-200c overexpression had an even more pronounced positive effect on apoptosis induced by proteasomal inhibition. Together, our data define miR-200c as a potentiator of bortezomib-induced cell death. At the same time, we show that miR-200c is a novel negative regulator of the pro-apoptotic Bcl-2 family member Noxa.

## Introduction

Death induced by the intrinsic mitochondrial pathway is initiated by perturbation of the mitochondrial membrane, and proceeds via release of cytochrome c and other apoptogenic factors from the intermembrane space of this organelle. This process is tightly regulated by the anti- and pro-apoptotic members of the Bcl-2 family [1]. Cytochrome c release in response to various types of cellular stress is suggested to occur via pores formed by homo and hetero-oligomers of the pro-apoptotic Bcl-2 family members Bak and Bax [2]. The actual ratio of anti- to pro-apoptotic Bcl-2 family members constitutes a sensor and sets the threshold of susceptibility to apoptosis for the cell. That the relative abundance of anti-apoptotic and pro-apoptotic regulators also critically influences tumorigenesis is illustrated by the recurring perturbation of this balance in cancer [3]. Consequently, the expression of Bcl-2 family members is normally tightly regulated at multiple levels including transcriptional activation and proteasomal degradation [1].

In recent years, microRNAs have emerged as important regulators of gene expression. MicroRNAs are 21–23 bp long non-coding RNAs that function mainly through targeting the 3′UTR of specific genes and thereby inhibiting the translation of their encoded protein or degrading the target mRNA [4], [5]. With their ability to regulate multiple genes simultaneously, microRNAs have fundamental roles in such diverse processes as proliferation, apoptosis and differentiation. Furthermore, many microRNAs, such as those of the miR-15, let-7, or miR-17 families have been shown to be deregulated in cancer, resulting in the altered expression of target genes important for tumor development [6].

Some Bcl-2 family members have been shown to be regulated by microRNAs, such as Bcl-2, which is regulated by miR-15/16 and miR-148a, [7], [8], [9] and Mcl-1, which is regulated by miR-29 [10]. However, for many of the Bcl-2 family members, including the pro-apoptotic p53 target gene Noxa, it is unknown whether microRNA regulation takes place. Like other BH3-only proteins, Noxa has the capacity to bind and neutralize pro-survival Bcl-2 family members. However, it has a restricted binding pattern and mainly interacts with Mcl-1 [11]. Among other things, this interaction leads to proteasomal degradation of Mcl-1 [12], [13], which in turn has been shown to be a prerequisite for apoptosis in response to for example UV irradiation [14].

**Figure 1:**
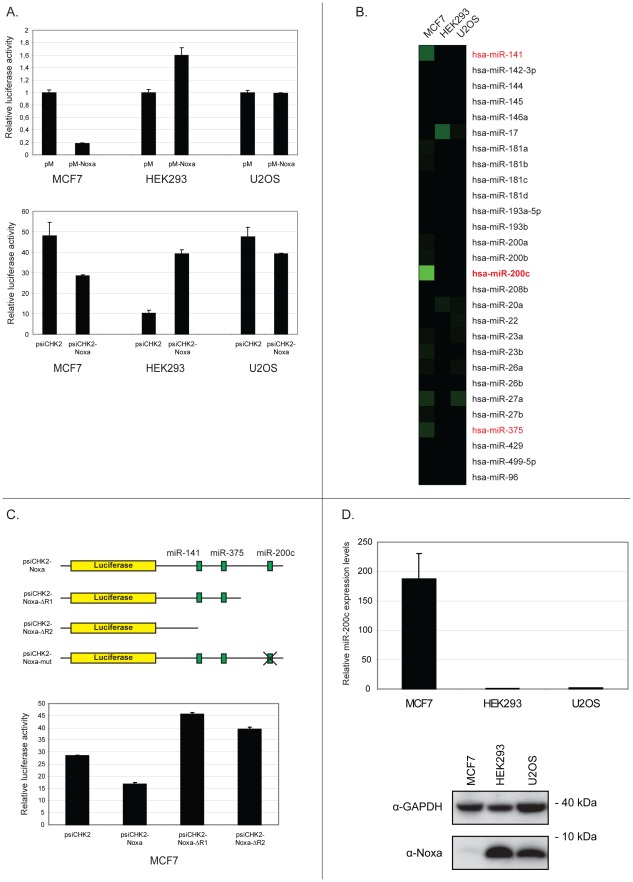
MiR-200c is a candidate Noxa-regulating microRNA. (**A**) The Noxa 3′UTR is repressed in MCF7 cells. The pMIR-REPORT (*upper panel*) and psiCHECK2 (*lower panel*) vectors with the full length Noxa 3′UTR downstream of luciferase or empty vector controls were introduced into the indicated cell lines. Luciferase activity was normalized to the activity of an external Renilla luciferase plasmid (*upper panel*) or to an internal Firefly luciferase (*lower panel*). (**B**) Expression profiling of microRNAs predicted to target the Noxa 3′UTR. (**C**) The repressive element is located in the distal part of the Noxa 3′UTR. The full length Noxa 3′UTR luciferase plasmid or the indicated deletion mutants were introduced into MCF7 cells and luciferase activity was measured as in (A). A schematic representation of the different 3′UTR constructs used in this study is also shown. The location of target sites of the three Noxa-regulating candidates is included in the scheme. (**D**) Expression of Noxa inversely correlates with that of miR-200c. MiR-200c expression was determined by qRT-PCR analysis in the indicated cell lines. Expression was normalized to that of the small nucleolar RNA RNU48 (*upper panel*). Protein extracts were prepared in parallel and subjected to immunoblotting for endogenous Noxa (*lower panel)*. GAPDH was used as a loading control. Protein size in kilodaltons (kDa) is also shown.

Given the ability of Noxa to fine-tune apoptotic signaling in response to various stimuli, and that Noxa protein induction is necessary for cell death to occur following treatment with some cytotoxic cancer drugs [13], we set out to investigate if Noxa is regulated by microRNAs. Any given gene is generally predicted to be regulated by many different microRNAs [15]. One major obstacle in microRNA research is that the numerous bioinformatic tools available for target prediction invariably give a large set of false positive results [16]. Therefore, we made use of a luciferase-based screening method to pick out the most relevant microRNAs that target Noxa. Cloning the 3′UTR of Noxa downstream of a luciferase reporter and introducing this construct into cells allowed us to determine to what degree the reporter activity is repressed in different tissues. This analysis was then complemented with luciferase experiments using deletion constructs that pinpointed the critical regulatory part(s) of the 3′UTR. Finally, the combined results were then compared with existing microRNA expression profiling data [17] to identify candidate microRNA(s) that might account for the differential luciferase activity. Using this screening system we identified miR-200c as a new regulator of Noxa. MiR-200c was shown to repress both basal and stress-induced Noxa protein expression. Surprisingly, enforced miR-200c expression at the same time led to increased bortezomib-induced apoptosis. This apparent discrepancy was reconciled by the finding that in cells devoid of Noxa expression, miR-200c caused an even greater increase in apoptosis. These data suggest that miR-200c potentiates apoptosis induced by proteasomal inhibitors but that it concomitantly represses Noxa which leads to an attenuated apoptotic induction.

**Figure 2:**
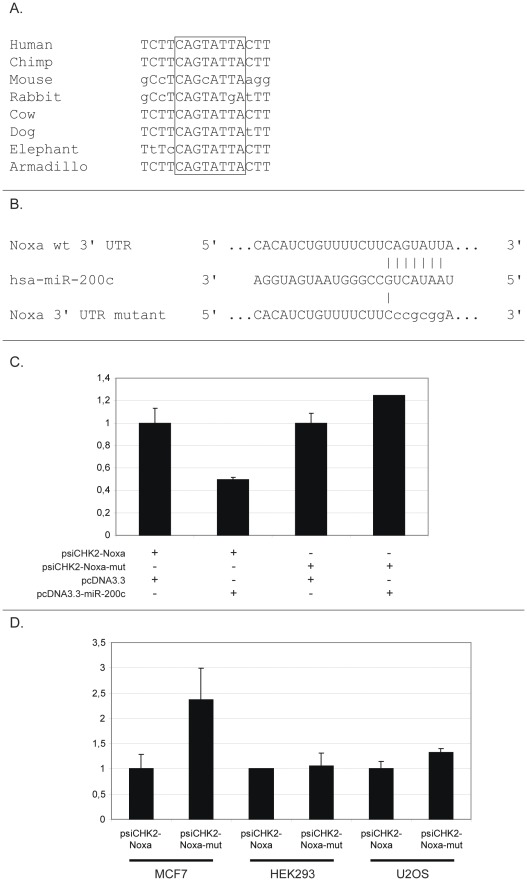
MiR-200c directly targets the Noxa 3′UTR. (**A**) The extent of evolutionary conservation of the predicted miR-200c target site in the Noxa 3′UTR is shown. Outlined box demarks region complimentary to the seed sequence of miR-200c. (**B**) Specific mutations introduced at the miR-200c target position in the Noxa 3′UTR reporter. (**C**) The indicated constructs were introduced into HEK293 cells and luciferase activity was measured. The luciferase ratio between the Renilla and Firefly of the empty vector transfection was adjusted to 1. (**D**) Luciferase reporter experiments were performed as in Figure 1A with the wild-type Noxa 3′UTR or the seed sequence mutant reporter constructs. The luciferase ratio between the Renilla and Firefly of the wild type Noxa 3′UTR vector transfection was adjusted to 1.

The data in this study define miR-200c as a novel regulator of Noxa and more generally show that microRNA-induced phenotypes must always be viewed as the complex results of a large number of occurring individual microRNA:mRNA target interactions.

## Materials and Methods

### Constructs, Inhibitors and Cloning

The miR-200c cluster expression construct was created by nested PCR cloning using the pcDNA™ 3.3 TOPO® TA Cloning Kit (Invitrogen). As first round primers miR200c-F1 (5′-GATGAGGGTGGGTAAATCGG-3′) and miR200c-R1 (5′-AGGGACTGGGTTAATCTGCT-3′) were used. Nested primers were miR200c-F2 (5′-CTGCTTGGACTGCAACCT-3′) and miR200c-R2 (5′-GTCTCCTTCCCATTGTTCCC-3′). The sequence of the cloned insert was verified in its entire length by sequencing. qRT-PCR was used to confirm that miR-200c was properly processed following plasmid transfection. The empty pcDNA-3.3-TOPO vector was used as a negative control in all transfection experiments. The full length (approximately 1.6 kb) Noxa 3′UTR was cloned using the primers Noxa-UTR-F1 (5′-CGAGTGTGCTACTCAACTCAG-3′) and Noxa-UTR-R1 (5′- CAGAGGATGTCTGCTGATGG-3′) as first round primers and Noxa-UTR-F2(XhoI) (5′-**CTCGAGTGA**CTGCATCAAAAACTTGCATGA-3′) and Noxa-UTR-R2 (5′- CACAGAAAAGAACAGTGAAAACT-3′) as nested primers. The XhoI restriction site in the Noxa-UTR-F2(XhoI) primer allowed for directional subcloning into the pMIR-REPORT™ (Ambion) and psiCHECK™-2 (Promega) luciferase vectors. The Noxa 3′UTR deletion mutants psiCHK2-Noxa-ΔR1 and psiCHK2-Noxa-ΔR2 were created by using Noxa-UTR-R1(NotI) ((5′-**GCGGCCGC**GGAACTTTCATTCTAGATGAG-3′) and Noxa-UTR-R2(NotI) (5′-**GCGGCCGC**CTGAGTATAACATTTCAGT-3′) respectively as reverse primers for cloning. The NotI restriction site in the reverse primers allowed for directional subcloning into the psiCHECK™-2 vector. The seed sequence mutant psiCHK2-Noxa-Mut construct was created using the QuikChange II Site Directed Mutagenesis Kit (Stratagene). The Noxa overexpression plasmid was constructed by amplifying the Noxa coding region from a pool of cell line cDNAs using the KAPA2G Fast PCR mix (KAPABiosystems) with the primers Noxa-F (GGACTGTTCGTGTTCAGCTC) and Noxa-R (TTTCCATCTAAAGTGACTACAACC). Amplicons were gel-purified and cloned into pcDNA3.3 using the pcDNA™ 3.3 TOPO® TA Cloning Kit (Invitrogen). The wild type sequence of the Noxa open reading frame in the construct was verified by sequencing.

**Figure 3:**
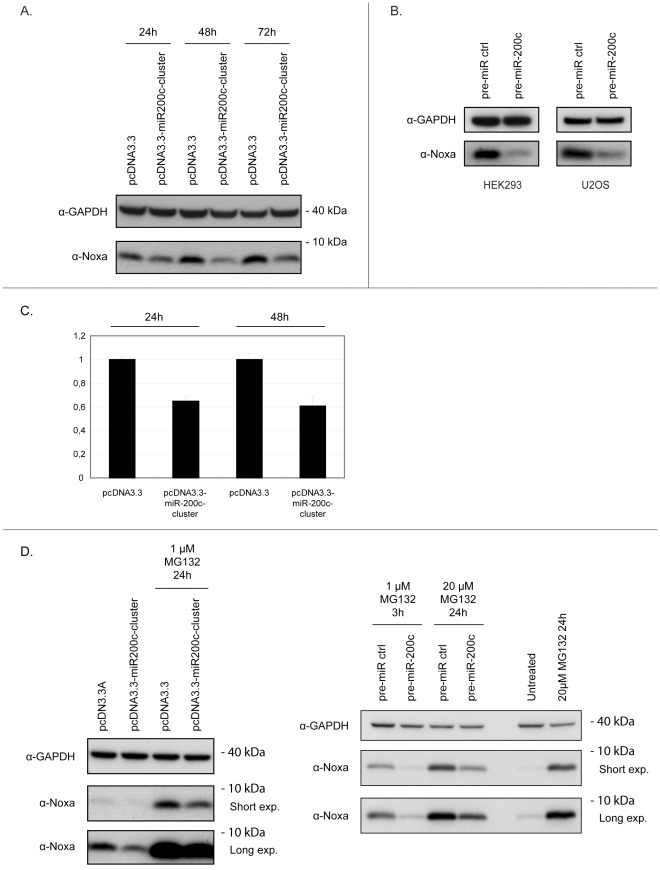
MiR-200c represses Noxa protein under both normal conditions and during cellular stress. (**A**) HEK293 cells were transfected with a plasmid encoding the miR-200c microRNA cluster or an empty vector control. Cells were collected at indicated timepoints, whole cell extracts were prepared and subjected to immunblotting for Noxa. (**B**) The indicated cell lines were transfected with pre-miR-200c or pre-miR-control oligos for 48 hours, whole cell extracts were prepared and analyzed for Noxa protein expression. (**C**) HEK293 cells were transfected with the indicated expression constructs for 24 and 48 hours respectively, and Noxa mRNA expression was determined by qRT-PCR analysis. Noxa mRNA expression was normalized to that of GAPDH. (**D**) HEK293 cells were transfected with indicated expression constructs (*left panel*) or oligonucleotides (*right panel*). 24 hours post-transfection, cells were treated with the indicated concentration of MG132 for an additional 3 or 24 hours, and processed for Noxa immunoblotting as in (A). GAPDH was used as a loading control in all immunoblotting experiments. Protein size in kilodaltons (kDa) is also shown.

### Oligonucleotides and Chemicals

Pre-miR-control (AM17110) and pre-miR-200c (PM11714) were purchased from Ambion. The miR-200c anti-miR™ inhibitor (AM11714) and anti-miR™ Negative Control #1 (AM17010) were obtained from Ambion. Noxa siRNA oligos (5′-GUAAUUAUUGACACAUUUC-3′) and control oligos targeting green fluorescent protein (5′-CAAGCUGACCCUGAAGUUC-3′) were purchased from Eurofins MWG and have been described previously [18], [19]. Bortezomib (Velcade®) was from Janssen-Cilag. MG132 was obtained from Calbiochem. Doxorubicin was purchased from MEDA AB.

**Figure 4:**
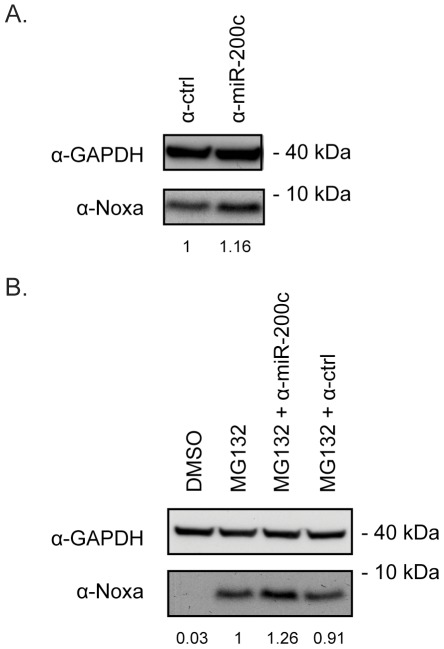
Noxa is repressed by miR-200c under normal conditions. (**A**) MCF7 cells were treated with miR-200c-specific inhibitors or control inhibitors for 48 hours and Noxa protein was analyzed as in Figure 3A. (**B**) MCF7 cells were treated with miR-200c-specific inhibitors or control inhibitors. 24 hours post-transfection, cells were treated with 1 µM MG132 for an additional 24 hours, and processed for Noxa immunoblotting. Bands were quantified using ImageJ software by normalizing the intensities for Noxa with GAPDH. Protein size in kilodaltons (kDa) is also shown.

### Bioinformatic Sequence Analysis

MiRNAs potentially targeting the 3′UTR of Noxa were predicted using TargetScan (http://www.targetscan.org/) miRanda (http://www.microrna.org/microrna/home.do) and PicTar (http://pictar.mdc-berlin.de/). In order to qualify as a possible Noxa regulator, a microRNA had to be predicted to have at least one microRNA target site in the Noxa 3′UTR according to at least two out of three prediction algorithms. MicroRNA expression data was obtained from microRNA.org (http://www.microrna.org/).

**Figure 5:**
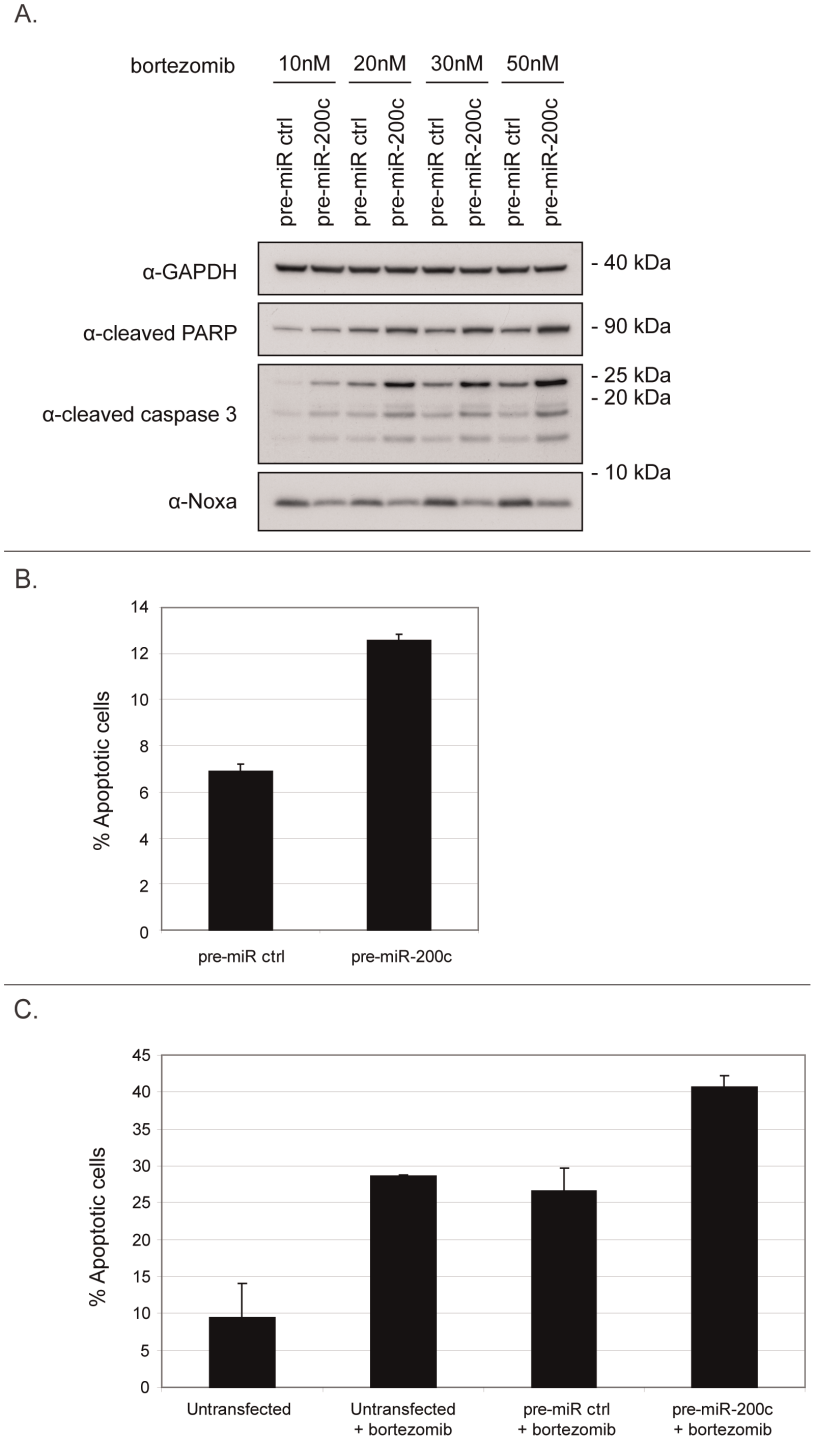
MiR-200c potentiates proteasome inhibitor-mediated cell death. (**A**) HCT116 cells were transfected with pre-miR-200c or pre-miR-control oligos for 24 hours, treated with indicated concentrations of bortezomib for an additional 24 hours and processed for immunoblotting for GAPDH, Noxa, cleaved PARP and cleaved caspase 3. Protein size in kilodaltons (kDa) is also shown. (**B**) HCT116 cells were transfected with pre-miR-200c or pre-miR-control oligos for 48 hours and apoptosis was assessed by Annexin V/PI staining and FACS analysis. (**C**) HCT116 cells were treated with 20 nM bortezomib as in (A) and apoptosis was assessed by Annexin V/PI staining and FACS analysis. Graphs show the mean of percentages of Annexin V-positive cells, including PI-positive and PI-negative, from three independent experiments.

### Cell Lines, Transfections and Treatments

MCF7, HEK293 and U2OS cells, all originally obtained from American Type Culture Collection (ATCC) (http://www.lgcstandards-atcc.org/), were maintained in Dulbecco’s Modified Medium (DMEM/High Modified, Hyclone) containing 10% FBS, 2 mM glutamine, 100 U/ml penicillin, and 100 g/ml streptomycin. HCT116 cells were grown in McCoy’s 5A medium (with L-glutamine, GIBCO) supplemented with 10% FBS, 100 U/ml penicillin, and 100 g/ml streptomycin. HCT116 wild type and *DICER1* knockout cells [20] were kindly provided by B Vogelstein and K Kinzler (John Hopkins University and Howard Hughes Medical Institute). siRNA oligos and pre-miRNA oligos were transfected using HiPerFect reagent (Qiagen), according to the manufacturer’s instructions. Transfections of miRNA inhibitors and cotransfections of plasmids and pre-miRNA oligos were performed using Lipofectamine 2000 (Invitrogen). For plasmid transfection experiments, cells were transfected with TransIT®-LT1 Transfection Reagent (Mirus) or GeneJuice® Transfection Reagent (Novagen) according to the manufacturers’ protocol. Typically, 1.0 µg plasmid, 100 nM siRNA oligos, 50 nM pre-miRNA oligos or 200 nM miRNA inhibitors were transfected per 6-well, if not otherwise stated. Generally, cells were transfected, incubated for 24 hours and then treated with the indicated drug for the indicated time period.

**Figure 6:**
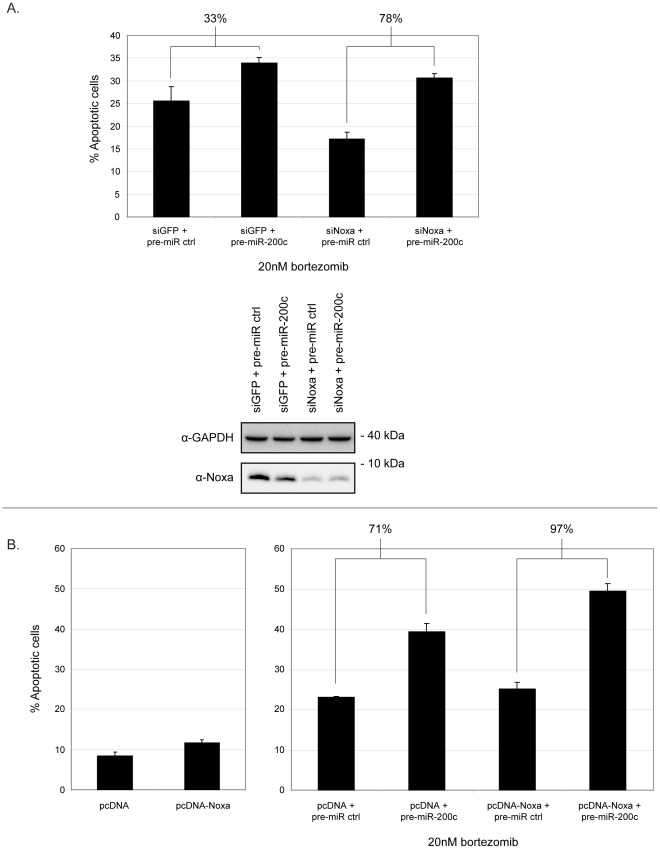
Noxa attenuates the proapoptotic effect of miR-200c. (**A**) HCT116 cells were transfected with pre-miR-200c or pre-miR-control oligos together with Noxa or GFP control siRNA oligos for 24 hours. Cells were treated with 20 nM bortezomib for an additional 24 hours and apoptosis was assessed by Annexin V/PI staining and FACS (*upper panel*). Cells were also collected and processed for immunoblotting for GAPDH and Noxa to demonstrate efficiency of siRNA knockdown (*lower panel*). Protein size in kilodaltons (kDa) is shown. (**B**) HCT116 cells were transfected with the indicated constructs and oligos. Samples were either left untreated (*left panel*) or treated with 20 nM bortezomib (*right panel*) and were subsequently analyzed as in (A). Graphs show the mean of percentages of Annexin V-positive cells, including PI-positive and PI-negative, from three independent experiments. Percentage values above graphs show fold increase in apoptosis when comparing indicated samples.

### Western Blot Analysis and Antibodies

Cells were lysed in NP-40 buffer (150 mM NaCl, 1% Nonidet P-40, 50 mM Tris pH 8.0) containing protease and phophatase inhibitors (Roche) and western blot analysis was performed as described previously [21]. The following primary antibodies were used: mouse monoclonal against Noxa (Calbiochem), rabbit polyclonal against GAPDH (Abcam), rabbit polyclonal against cleaved caspase 3 (Asp175, Cell Signaling) and rabbit polyclonal against cleaved PARP (Asp214, Cell Signaling). As secondary antibodies HRP-conjugated anti-rabbit and anti-mouse antibodies (Cell Signaling) were used.

### Quantitative Real-time PCR (qRT-PCR)

Total RNA was extracted from indicated cell lines using Trizol reagent (Life Technologies) and reverse transcribed using Superscript II (Invitrogen) with random primers. Real-time PCR for Noxa (primers: Noxa-F 5′-ACTGTTCGTGTTCAGCTC-3′ and Noxa-R 5′-GTAGCACACTCGACTTCC-3′, [22]) was performed using the Power SYBR® Green PCR Master Mix (Applied Biosystems) in triplicate measurements on a 7900 HT Fast Real-time PCR system (Applied Biosystems). Samples were quantified using three point standard curves for each primer pair. Values were normalized to the relative quantity of GAPDH (primers: GAPDH-F 5′-AGCCGAGCCACATCGCT-3′ and GAPDH-R 5′-GCAACAATATCCACTTTACCAGAGT-3′). MiR-200c was detected with Applied Biosystems TaqMan miRNA assays (Product ID 002300). RNU48 (Product ID 001006) was used as an internal standard. Relative quantification of microRNA expression level was performed using the comparative Ct method, 2−(ΔCt_sample_–ΔCt_control_).

### Luciferase Reporter Assays

Luciferase reporter assays were carried out using the Dual-Luciferase Reporter Assay System (Promega) as previously described [18]. PsiCHECK™-2 or pMIR-REPORT™ luciferase vectors containing the full-length, wild-type Noxa 3′UTR or mutant versions were used for transfections. The psiCHECK™-2 vector encodes an additional Firefly luciferase that allows for normalization of transfection. With the pMIR-REPORT™ vector a separate Renilla luciferase plasmid was cotransfected for normalization. 20 ng of respective 3′UTR reporter plasmid was cotransfected with 480 ng microRNA-encoding plasmid in 24-well plates.

### Assessment of Apoptosis by Annexin V and Propidium Iodide Stainings

Redistribution of plasma membrane phosphatidyl serines was assessed using Annexin V FLUOS (Boehringer Mannheim) according to the manufacturer’s protocol. Annexin V and propidium iodide (PI) stainings were performed and analyzed using a FACScalibur flow cytometer (Becton Dickinson) with Cell Quest Software as previously described [23].

## Results

### Luciferase-based Screening Identifies miR-200c as a Potential Regulator of Noxa

In order to evaluate whether Noxa is regulated by microRNAs, we first analyzed Noxa protein levels in HCT116 cells lacking *DICER1* (HCT116*^DICER1^* ^−/−^), an RNase III enzyme required for microRNA processing. Noxa protein was increased in HCT116*^DICER1^* ^−/−^ as compared to wild type cells, suggesting that Noxa expression indeed is under the influence of microRNA regulation (Figure S1). In order to identify which microRNAs that regulate Noxa, we first cloned the entire Noxa 3′UTR into the pMIR-REPORT vector downstream of luciferase. This vector was introduced into MCF7, HEK293 and U2OS cells and luciferase activity was measured. As can be seen in Figure 1A, luciferase expression was potently reduced in MCF7 cells while no repression was observed in the other cell lines. In order to exclude the possibility that this difference simply reflected differential usage of the promoter driving luciferase in the different cell lines, the Noxa 3′UTR was also cloned into the psiCHECK2 luciferase vector. Using this construct, a similar result was obtained (Figure 1A, *lower panel*). This raised the possibility that one or several microRNAs that are expressed in MCF7 cells, but not in HEK293 or U2OS cells, regulate Noxa expression. We proceeded to compile the expression of all microRNAs predicted to target Noxa according to the TargetScan, PicTar and miRanda algorithms (Figure 1B and Materials and Methods). Notably, miR-141, miR-200c and miR-375 displayed moderate to high levels of expression in MCF7 cells with little or no expression in HEK293 and U2OS. In order to examine the relative impact of these three microRNAs on Noxa regulation, luciferase reporter truncation mutants with progressively shorter UTRs were created and introduced into MCF7 cells. Figure 1C shows that luciferase activity was restored already with the longest deletion mutant, indicating that the repressive element is located in the distal 0.5 kb of the Noxa 3′UTR. Of the three candidate microRNAs, only miR-200c has a predicted target site in the distal part of the Noxa 3′UTR (Figure 1C). These results strongly suggest that miR-200c regulates the Noxa 3′UTR. Finally, the differential expression of miR-200c in the three cell lines was confirmed by qRT-PCR and was found to inversely correlate with that of endogenous Noxa protein expression (Figure 1D).

In conclusion, by making use of a luciferase-based screening method together with microRNA expression profiling we could identify miR-200c as a high-probability Noxa-regulating microRNA.

### Noxa is a Direct Target of miR-200c

The Noxa 3′UTR contains one miR-200c target site that is evolutionarily conserved down to armadillo (*Dasypus novemcinctus*) (Figure 2A). In order to examine whether miR-200c regulates Noxa, we mutated the seed region of this site, which is thought to interfere with microRNA binding (Figure 2B). When cotransfecting the wild type Noxa 3′UTR luciferase vector and miR-200c, luciferase expression was decreased by around 50%. On the other hand, the mutated vector (psiCHK2-Noxa-Mut) was completely refractory to miR-200c-mediated repression (Figure 2C). These results indicate that miR-200c directly targets Noxa and that this regulation is dependent on one single target site located in the distal part of the Noxa 3′UTR. We next introduced the wild type and mutated vector respectively into the cell lines used for the initial screening. As can be seen in Figure 2D, a clear derepression was observed with the mutant construct in MCF7 cells (with high miR-200c expression), while little or no change in luciferase activity was seen in the other cell lines with low or absent miR-200c.

These results show that Noxa is a direct target of miR-200c and that miR-200c is a major determinant of 3′UTR-mediated Noxa regulation.

### miR-200c Downregulates Noxa Expression

We transfected HEK293 cells, which have low endogenous miR-200c expression levels, with a vector encoding the miR-200c cluster and analyzed Noxa protein levels at different timepoints following transfection. As seen in Figure 3A, miR-200c overexpression resulted in a clear downregulation of Noxa expression at all timepoints analyzed. MicroRNA qRT-PCR was used to confirm proper miR-200c processing following plasmid transfection (Figure S2). Since the miR-200c cluster encodes both miR-200c and miR-141, we also transfected a pre-miR-200c oligonucleotide to investigate whether miR-200c expression alone is sufficient to repress Noxa. Expression of the pre-miR-200c oligonucleotide caused a clear downregulation of Noxa in several cancer cell lines (Figure 3B and data not shown). MicroRNAs repress gene expression by promoting RNA degradation and, to a lesser extent, by inhibiting translation [5]. Overexpression of the miR-200c cluster led to a significant downregulation of Noxa mRNA levels as measured by qRT-PCR (Figure 3C). This suggests that miR-200c indeed causes mRNA degradation of Noxa. Under unstressed conditions, Noxa levels in cells are generally very low, but are known to increase under conditions of cellular stress [13]. Therefore, we assessed whether miR-200c can modulate Noxa levels when Noxa is induced by proteasomal inhibition. HEK293 cells were transfected with the miR-200c cluster or an empty control vector and subsequently treated with the proteasomal inhibitor MG132. As can be seen in Figure 3D, induction of Noxa protein was attenuated in cells with overexpressed miR-200c. Again, overexpression of the pre-miR-200c oligonucleotide resulted in a similar decrease in Noxa protein levels upon proteasomal inhibition (Figure 3D, *right panel*). This effect was not dependent on cell type as miR-200c-mediated repression of induced Noxa was evident also in HCT116 cells (Figure S3).

Together these results demonstrate that miR-200c can downregulate Noxa RNA and protein under both normal conditions and during cellular stress caused by proteasomal inhibition.

### Noxa is Repressed by miR-200c Under Normal Conditions

In order to further examine whether the miR-200c-Noxa interaction takes place when miR-200c is expressed at endogenous levels, we made use of an inhibitor that binds miR-200c and prevents it from binding to its targets. When introduced into MCF7 cells a small, but consistent, increase in Noxa protein levels was observed (Figure 4A and data not shown). A similar picture emerged when cells were treated with MG132, again confirming that the miR-200c-mediated regulation takes place both under normal conditions and when Noxa is induced by exogenous stress (Figure 4B). Taken together, these results suggest that Noxa is regulated by endogenous miR-200c.

### miR-200c Potentiates Proteasome Inhibitor-mediated Cell Death

Given the effect of miR-200c on Noxa, we hypothesized that it could modulate cellular sensitivity to apoptosis. We therefore evaluated the effect of miR-200c on apoptosis induced by the proteasome inhibitor bortezomib. This clinically used drug was chosen since it has been shown that Noxa induction is important for bortezomib-induced cell death [19], [24], [25]. Treatment of HCT116 cells with clinically relevant doses of bortezomib led to a time- and dose-dependent induction of Noxa protein (Figure S4). As can be seen in Figure 5A, overexpression of miR-200c in HCT116 cells treated with bortezomib led to a downregulation of Noxa at all doses. Surprisingly, at the same time miR-200c overexpression resulted in increased bortezomib-induced apoptosis as assessed by immunoblotting for cleaved caspase 3 and cleaved PARP (Figure 5A). In order to directly test how apoptosis induction is affected by miR-200c overexpression, Annexin V/PI staining was performed on HCT116 left untreated or treated with bortezomib. Again, in both cases miR-200c overexpression led to increased cell death, as compared to a scrambled pre-miR control oligonucleotide (Figure 5B–C). A similar result was obtained in the HEK293 cell line (data not shown). Also, this effect was not restricted to proteasome inhibition, as cells treated with the DNA-damaging drug doxorubicin showed increased apoptosis induction upon miR-200c overexpression as well (data not shown). Since the effects of miR-200c on Noxa and cell death induced by bortezomib apparently contradict one another, we went on to examine the effect of miR-200c on apoptosis in a setting without Noxa expression. Therefore, we knocked down Noxa expression in bortezomib-treated HCT116 cells using siRNA oligos. Knockdown of Noxa led to an expected decrease in both Noxa protein levels and proteasome inhibitor-induced apoptosis as measured by Annexin V/PI staining (Figure 6A). Interestingly, when Noxa was knocked down, miR-200c overexpression had an even more pronounced effect on apoptosis induction. Indeed, in cells transfected with control siRNA oligos, miR-200c overexpression led to a 33% increase in apoptosis, as compared to cells transfected with scrambled pre-miRs. In contrast, in cells with Noxa knocked down the increase in apoptosis was 78% (Figure 6A). To further investigate the relationship between miR-200c, Noxa and bortezomib-induced cell death, we went on to ectopically express a Noxa construct lacking the miR-200c target site (Figure S5). When Noxa was overexpressed in cells left untreated with bortezomib, only a minor effect on apoptosis could be observed (Figure 6B, *left panel*). However, overexpression of Noxa potentiated the positive effect of miR-200c on bortezomib-induced apoptosis (Figure 6B, 71% versus 97% increase in apoptosis), showing that artificially maintaining high Noxa levels in cells increases the pro-apoptotic effects of miR-200c even further. In summary, these data show that miR-200c sensitizes cells to bortezomib treatment. However, at the same time it represses Noxa, which leads to an attenuated bortezomib response.

## Discussion

In this study we identify and validate miR-200c as a regulator of the proapoptotic BH3-only member Noxa. Much is known regarding the transcriptional regulation of Noxa. Several types of cellular stress, such as DNA damage and hypoxia, lead to Noxa induction in both a p53-dependent and independent fashion [13]. However, nothing has so far been reported concerning possible microRNA regulation of Noxa. The identification of miR-200c as a Noxa regulator was facilitated by a methodology that combines a luciferase-based screening with mining of microRNA expression data (Figure 1). This method is broadly applicable to the identification of other microRNA:target interactions.

Obviously, other mechanisms than microRNAs exist that regulate gene expression through the 3′UTR. Several recent studies have demonstrated the importance of for example RNA-binding proteins in posttranscriptional gene regulation [26], [27], [28]. However, it has also been shown that in many cases there is extensive interplay between microRNAs and RNA-binding proteins [29]. For example, miR-16 is necessary for the regulated turnover of AU-rich element (ARE) containing mRNAs by the ARE-binding protein tristetraprolin [30]. The fact that microRNA-mediated gene repression makes up a substantial part of 3′UTR-mediated regulation was substantiated in a recent report investigating the impact or shortened 3′UTRs on oncogenic transformation. When isoforms of varying 3′UTR length of the IMP-1 oncogene were used in soft-agar colony formation assays, it was demonstrated that the shorter isoforms were more oncogenic than the longer ones. Importantly, this difference in transformation ability was mostly attributed to loss of miRNA targeting, since microRNA target site mutants yielded significantly enhanced transformation from the longer isoforms [31]. One advantage with our method is that one is not restricted to the cell lines used in the current study and it is of course straightforward to change and expand the selection of cell lines to a set that is optimal for a given target gene. Furthermore, as more expression data is emerging, especially given the amounts of information originating from the recently developed mass sequencing technologies, more and more tissues will be available for consideration. Using a set of broadly used cancer cell lines, the method allowed us to relatively quickly limit the number of possible candidates and eventually end up on a true microRNA:target interaction. The interaction was validated using a series of experiments. First, overexpression of miR-200c led to dramatically reduced Noxa levels in several cancer cell lines. Importantly, this regulation occurred both in unstressed cells and cells exposed to proteasomal inhibitors (Figures 3, 5 and S3). That miR-200c directly targets the 3′UTR of Noxa at a defined evolutionarily conserved site was established using luciferase assays (Figure 2A–C). Finally, with the help of specific miR-200c inhibitors we could show that Noxa is normally under repression from endogenous miR-200c (Figure 4).

The miR-200 family of microRNAs (also known as the miR-8 family) consists of 5 members (miR-200a, miR-200b, miR-200c, miR-141 and miR-429) expressed from two genomic locations. They can be subdivided into two major groups that differ slightly with regard to seed sequences and that have partly overlapping but distinct sets of targets [32]. Several studies have reported that the miR-200 microRNAs are potent regulators of epithelial-to-mesenchymal transition (EMT), a process that occurs during embryonic development, wound healing and cancer metastasis [33]. During EMT, epithelial cells acquire a more mesenchymal phenotype with increased motility and invasiveness and decreased cell-cell adhesion. A key event during this transition is the loss of the epithelial marker E-cadherin. MiR-200 family members have been shown to target the transcription factors ZEB1 and ZEB2 that normally repress the expression of, among other genes, E-cadherin and in this way miR-200 microRNAs help to maintain the cell in an epithelial state [33], [34], [35]. It is known that EMT is intimately linked to cancer development and that metastasizing cells undergo a process that is very similar to EMT. However, cancer cells can also undergo the reverse process, mesenchymal-to-epithelial transition (MET), when colonizing distant sites in the body following extravasation. In light of this it is perhaps not surprising that a complex picture emerges with regard to cancer and miR-200. While many tumor types, such as advanced breast cancer and clear cell carcinoma, show reduced miR-200 levels, some other malignancies instead display overexpressed miR-200 levels [33]. One speculative possibility is that downregulation of miR-200 occurs in some tumors when the cancer cells become invasive and that this is followed by miR-200 upregulation in distant metastases that undergo MET.

While the novel miR-200c target Noxa is dispensable for certain types of cell death, it is crucial for cell death in response to proteasomal inhibition [19], [24], [25], [36], [37], [38]. The proteasome inhibitor bortezomib (PS-341, Velcade®) has been demonstrated to be clinically beneficial in the treatment of multiple tumor types, including myeloma and mantle cell lymphoma [39], [40]. We therefore chose to study its impact in relation to miR-200c. The observed effects of miR-200c on Noxa and cell death induced by bortezomib and other agents might at first appear counterintuitive. Why would miR-200c potentiate apoptosis and repress Noxa at the same time? One possible reason is that is a matter of threshold. MiR-200c keeps Noxa in check to prevent premature or excessive apoptosis to occur. Once Noxa is induced to high enough levels following cellular stress, the interaction between miR-200c and Noxa becomes less relevant and other miR-200c targets play a more important role. Indeed, several targets have been described that could explain the pro-apoptotic effect of miR-200c, such as FAP-1 [41], PLCγ1 [32] and the above-mentioned ZEB1 [42]. In line with this, miR-200c has been described to potentiate apoptosis in response to CD95 signaling and microtubule-targeting agents [41], [43]. Also, it is possible that the miR-200c:Noxa interaction plays a more dominant role in other tissues or when Noxa is induced by other stimuli. One has to bear in mind that the phenotypic effect of a given microRNA is dictated by the sum total expression of all its potential targets. Yet another possibility would be that Noxa for some reason has an anti-apoptotic effect in our systems. However, without Noxa expression, the positive effect of miR-200c on apoptosis becomes even more pronounced (Figure 6A), indicating that Noxa indeed potentiates cell death induced by bortezomib. In line with this, ectopic expression of a Noxa construct unresponsive to miR-200c regulation led to potentiation of miR-200c-mediated apoptosis induction (Figure 6B). We thus have a situation where the pro-apoptotic effect of miR-200c is partially counteracted by its repressive effect on Noxa. Interestingly, a similar scenario was described for miR-128. It apparently induces apoptosis in HEK293T cells while at the same time it directly represses the pro-apoptotic Bax protein [44].

In conclusion, we have identified miR-200c as an apoptosis-regulating microRNA that represses Noxa. The data presented have implications for the understanding of apoptosis in general, and Noxa regulation in particular. Furthermore, it can also help explain the mechanism behind bortezomib resistance in different tumors.

## Supporting Information

Figure S1 **Protein extracts were prepared from HCT116** ***DICER1*** **wild type and knockout cells and analyzed for Noxa protein levels by immunoblotting.** GAPDH was used as a loading control.
(TIF)

Figure S2 **HEK293 cells were transfected with the miR-200c cluster expression vector or an empty vector control.** 48 hours post-transfection, cells were collected and processed for TaqMan qRT-PCR analysis. MiR-200c expression was normalized to that of the small nucleolar RNA RNU48 using the comparative Ct method. The expression level in mock-transfected cells is set to 1.
(TIF)

Figure S3 **HCT116 cells were transfected with pre-miR-200c or pre-miR-control oligos.** 24 hours post-transfection, cells were treated with the indicated concentrations of MG132 for an additional 24 hours, and processed for Noxa immunoblotting. GAPDH was used as a loading control. Protein size in kilodaltons (kDa) is also shown.
(TIF)

Figure S4 **Bortezomib induces a time- and dose-dependent increase in Noxa protein levels.** HCT116 cells were treated with increasing concentrations of bortezomib and analyzed for Noxa protein expression (*upper panel*). HCT116 cells were treated with 20 nM bortezomib, collected at the indicated timepoints and processed for immunoblotting for the indicated proteins (*lower panel*). While Noxa is induced already after three hours, cleaved PARP and caspase 3 immunoblots demonstrate that apoptosis is not properly executed until after 24 hours of treatment. GAPDH was used as a loading control. Protein size in kilodaltons (kDa) is also shown.
(TIF)

Figure S5 **HCT116 cells were transfected with empty vector (pcDNA) or with a Noxa overexpression construct (pcDNA-Noxa).** Protein extracts were analyzed for Noxa and GAPDH levels by immunoblotting. Protein size in kilodaltons (kDa) is also shown.
(TIF)
