# Supplementary figures and images for: MiR-200c Regulates Noxa Expression and Sensitivity to Proteasomal Inhibitors
Source: PLoS One. 2012 May 15;7(5):e36490. doi: 10.1371/journal.pone.0036490 (PMC3352905; doi:10.1371/journal.pone.0036490)

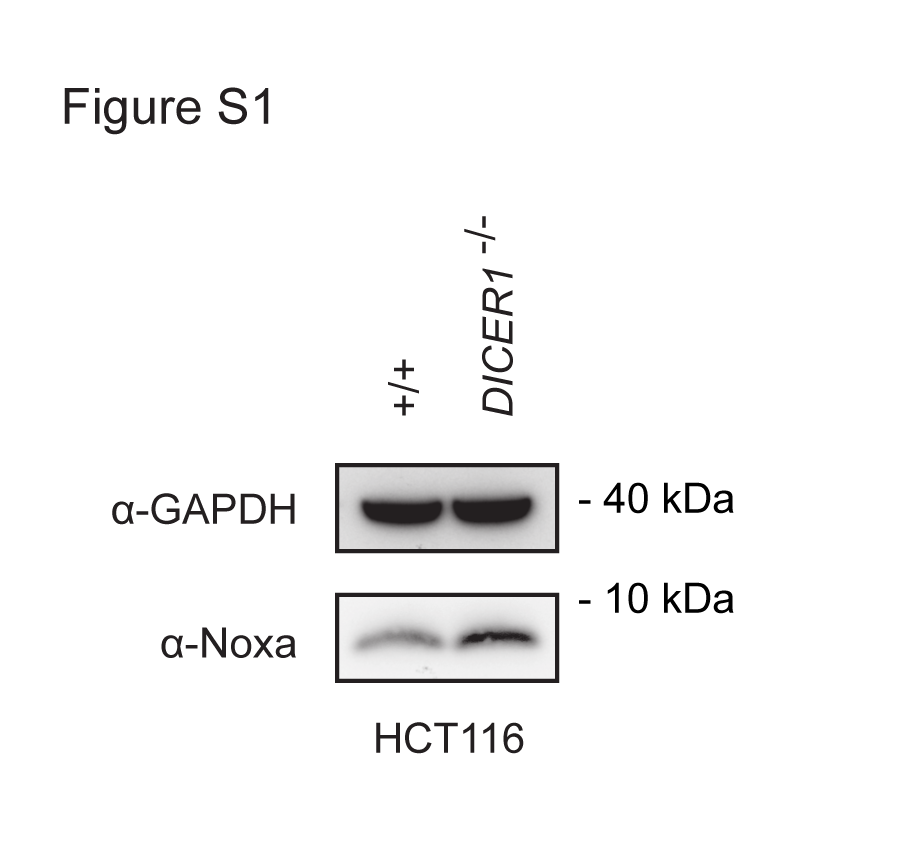

Supplement: Figure S1 — Protein extracts were prepared from HCT116 DICER1 wild type and knockout cells and analyzed for Noxa protein levels by immunoblotting. GAPDH was used as a loading control. (TIF) [file pone.0036490.s001.tif]

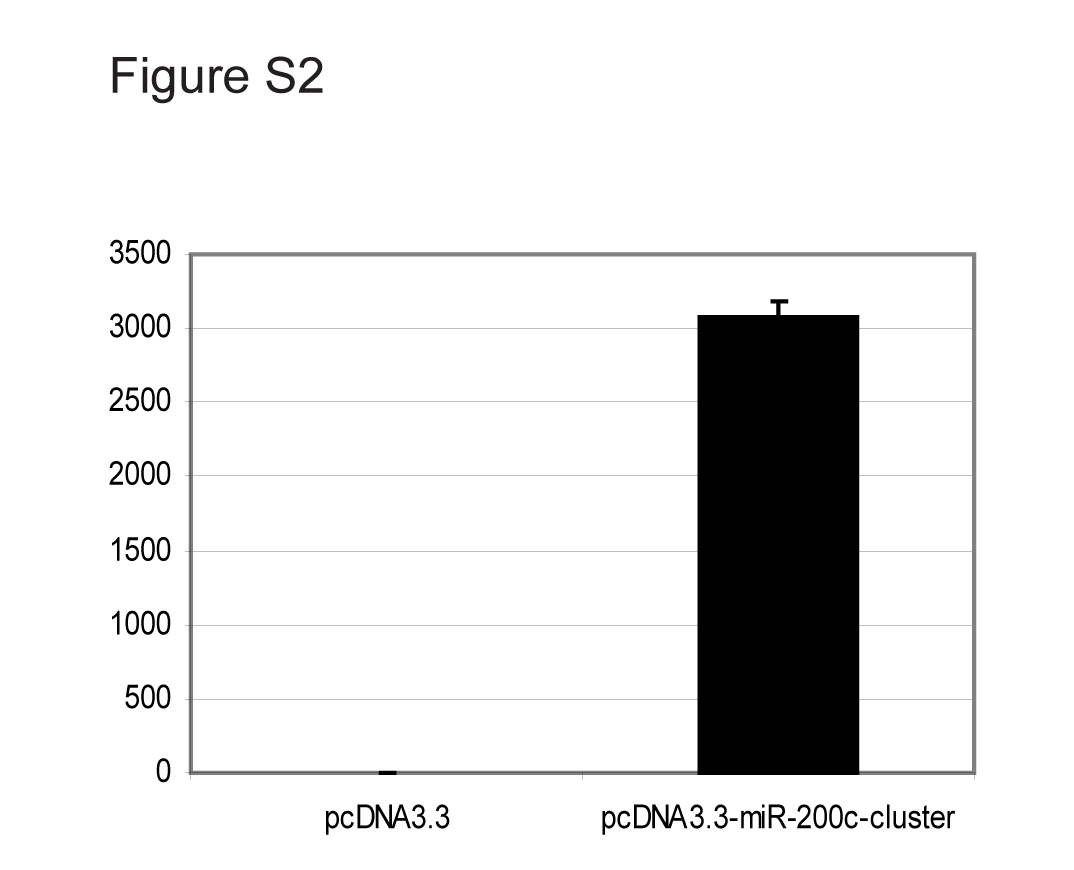

Supplement: Figure S2 — HEK293 cells were transfected with the miR-200c cluster expression vector or an empty vector control. 48 hours post-transfection, cells were collected and processed for TaqMan qRT-PCR analysis. MiR-200c expression was normalized to that of the small nucleolar RNA RNU48 using the comparative Ct method. The expression level in mock-transfected cells is set to 1. (TIF) [file pone.0036490.s002.tif]

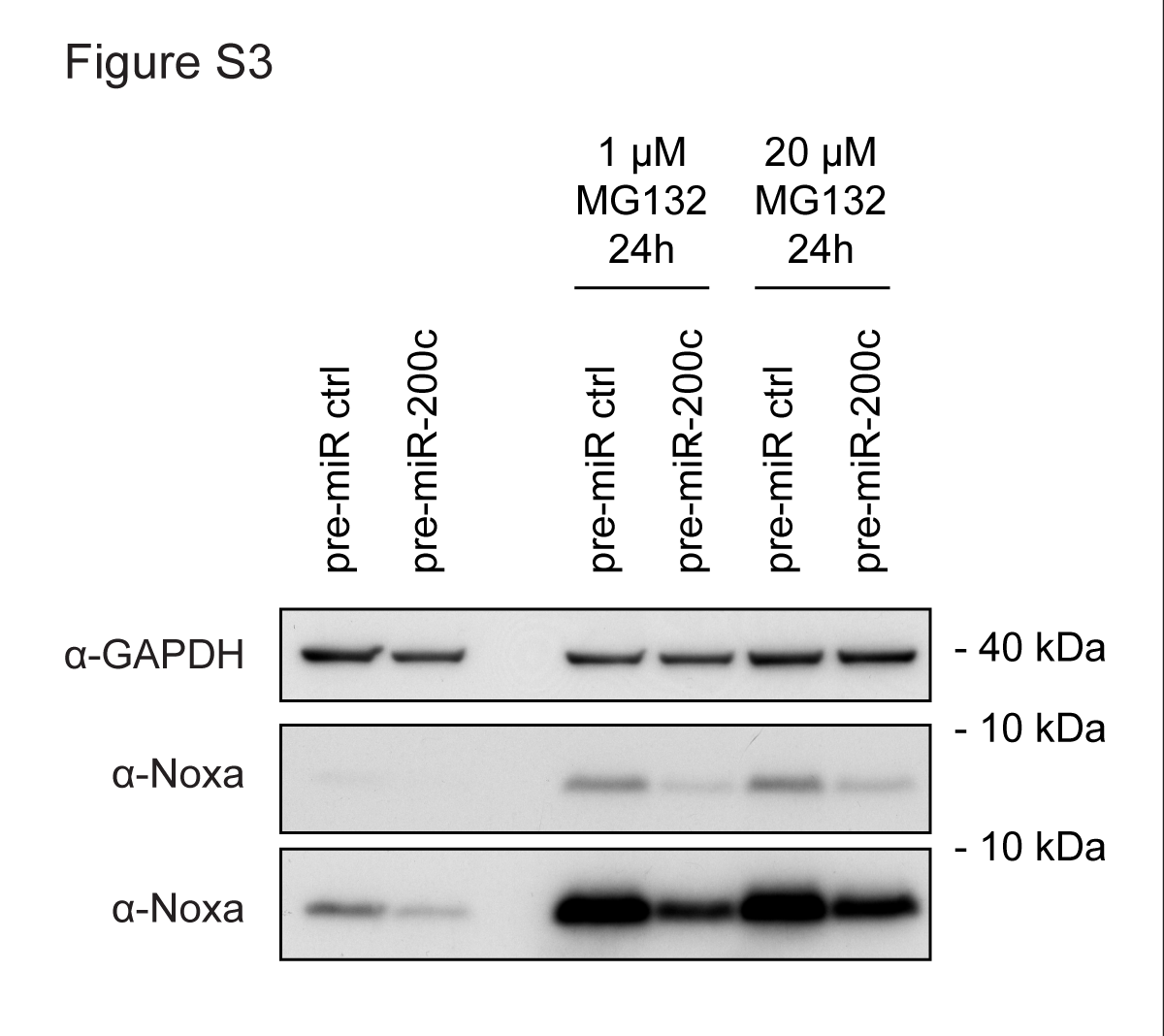

Supplement: Figure S3 — HCT116 cells were transfected with pre-miR-200c or pre-miR-control oligos. 24 hours post-transfection, cells were treated with the indicated concentrations of MG132 for an additional 24 hours, and processed for Noxa immunoblotting. GAPDH was used as a loading control. Protein size in kilodaltons (kDa) is also shown. (TIF) [file pone.0036490.s003.tif]

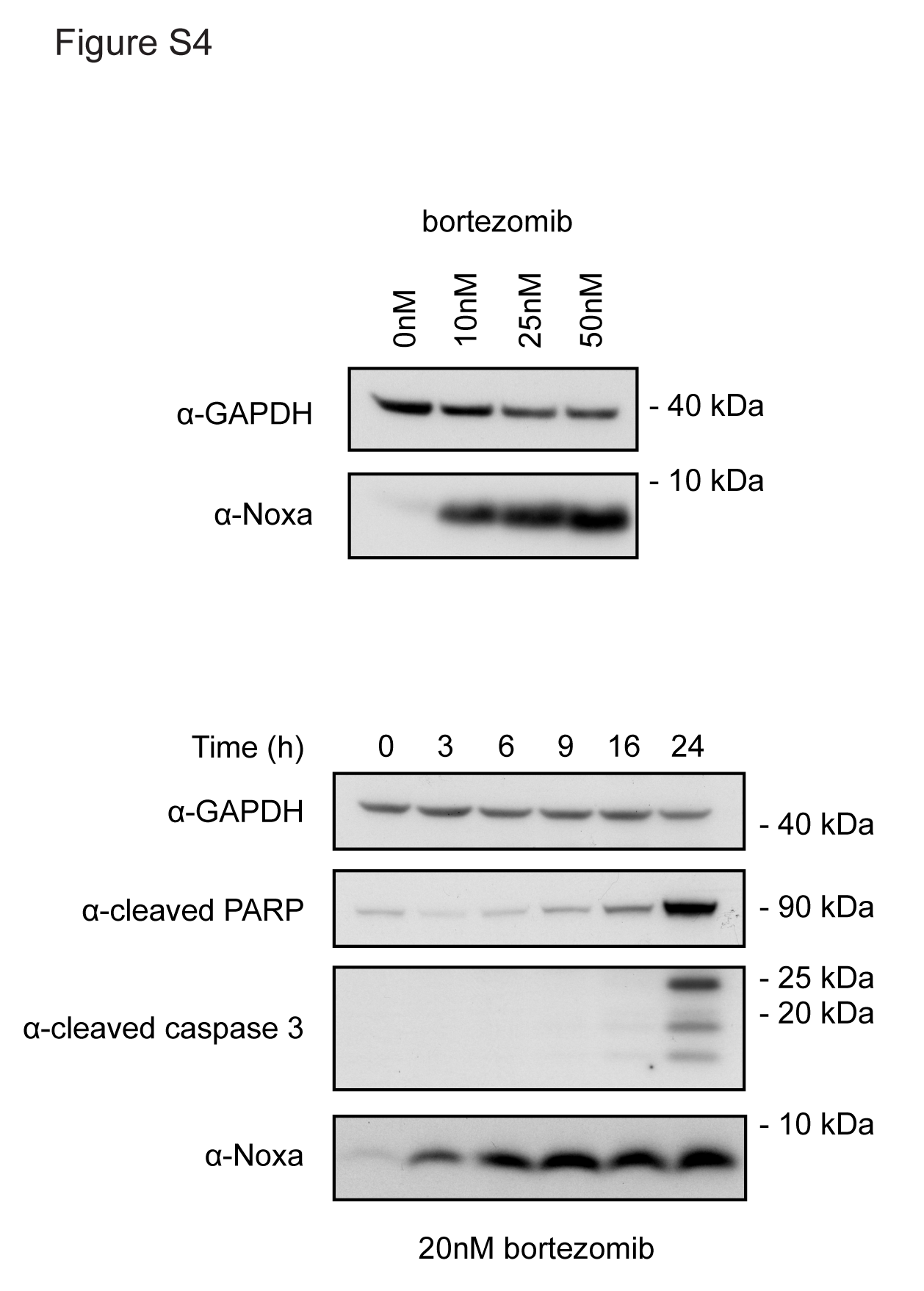

Supplement: Figure S4 — Bortezomib induces a time- and dose-dependent increase in Noxa protein levels. HCT116 cells were treated with increasing concentrations of bortezomib and analyzed for Noxa protein expression (upper panel). HCT116 cells were treated with 20 nM bortezomib, collected at the indicated timepoints and processed for immunoblotting for the indicated proteins (lower panel). While Noxa is induced already after three hours, cleaved PARP and caspase 3 immunoblots demonstrate that apoptosis is not properly executed until after 24 hours of treatment. GAPDH was used as a loading control. Protein size in kilodaltons (kDa) is also shown. (TIF) [file pone.0036490.s004.tif]

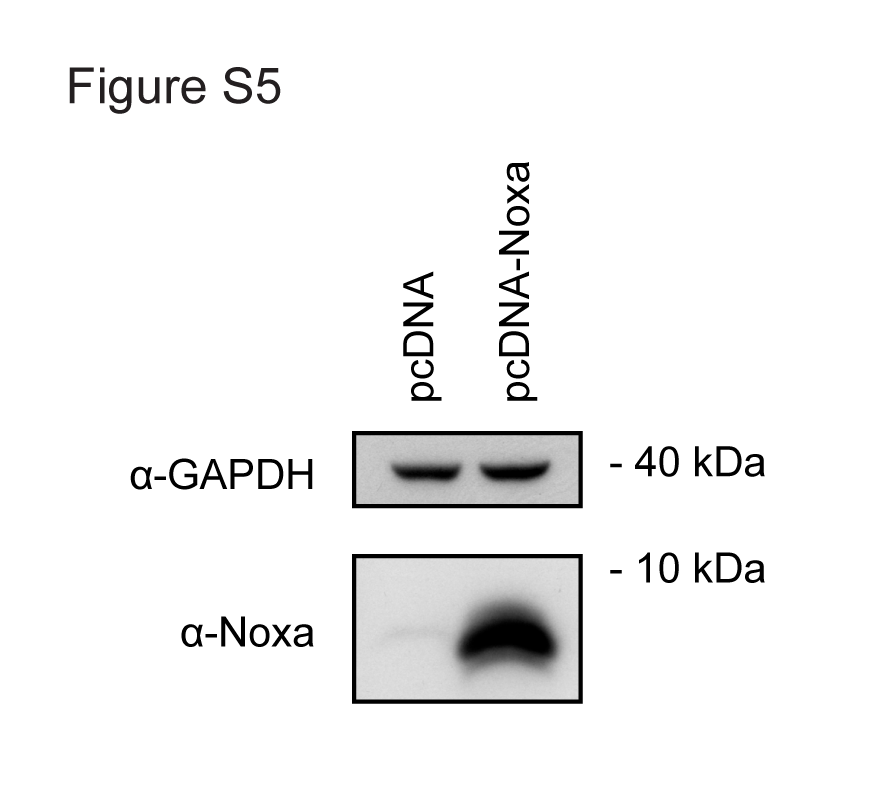

Supplement: Figure S5 — HCT116 cells were transfected with empty vector (pcDNA) or with a Noxa overexpression construct (pcDNA-Noxa). Protein extracts were analyzed for Noxa and GAPDH levels by immunoblotting. Protein size in kilodaltons (kDa) is also shown. (TIF) [file pone.0036490.s005.tif]
